# Intake of Lutein-Rich Vegetables Is Associated with Higher Levels of Physical Activity

**DOI:** 10.3390/nu7095378

**Published:** 2015-09-18

**Authors:** Georgina Crichton, Merrill Elias, Ala’a Alkerwi, Jonathon Buckley

**Affiliations:** 1 Alliance for Research in Exercise, Nutrition and Activity (ARENA), Sansom Institute for Health Research, University of South Australia, Adelaide 5001, Australia; E-Mail: Jon.Buckley@unisa.edu.au; 2 Department of Psychology, University of Maine, Orono, ME 04469, USA; E-Mail: mfelias@maine.edu; 3 Graduate School of Biomedical Sciences and Engineering, University of Maine, Orono, ME 04469, USA; 4 Luxembourg Institute of Health L.I.H. (formerly Centre de Recherche Public Santé), Grand-Duchy of Luxembourg, Strassen L-1445, Luxembourg; E-Mail: Alaa.Alkerwi@lih.lu

**Keywords:** lutein, vegetables, physical activity

## Abstract

Levels of physical inactivity, a major contributor to burden of disease, are high in many countries. Some preliminary research suggests that circulating lutein concentrations are associated with high levels of physical activity (PA). We aimed to assess whether the intake of lutein-containing foods, including vegetables and eggs, is associated with levels of PA in two studies conducted in different countries. Dietary data and PA data collected from participants in two cross-sectional studies: the Maine-Syracuse Longitudinal Study (MSLS), conducted in Central New York, USA (*n* = 972), and the Observation of Cardiovascular Risk Factors in Luxembourg Study (ORISCAV-LUX) (*n* = 1331) were analyzed. Higher intakes of lutein containing foods, including green leafy vegetables, were associated with higher levels of PA in both study sites. Increasing the consumption of lutein-rich foods may have the potential to impact positively on levels of PA. This needs to be further explored in randomized controlled trials.

## 1. Introduction

Levels of physical inactivity in the United States, Australia, and other countries are high. Approximately one-third of American adults do no physical activity (PA) [1,2]. Physical inactivity is a major contributor to burden of disease, predisposing to major chronic diseases including cardiovascular disease (CVD) [3], type 2 diabetes [4], certain cancers [5], dementia, and depression [6,7]. CVD accounts for approximately one of every three deaths in the United States, and is estimated to cost $312 billion annually [8]. As in the United States, CVD is the number one cause of mortality in Luxembourg [9]. Together these diseases present a huge public health cost, and the burden of disease is likely to continue to grow as the population ages and becomes less active.

Even modest increases in PA in previously inactive adults can substantially reduce the risk of chronic disease [3] and all-cause mortality [10]. These reductions in disease risk are achieved through a variety of mechanisms, including contributing to a healthy body weight, reducing inflammation, improving vascular function, reducing DNA damage and promoting DNA repair [11].

Lutein is one of 600 known naturally occurring carotenoids. Carotenoids are found in foods such as eggs, fruit, and vegetables, in particular green leafy vegetables. High plasma carotenoid concentrations have been associated with a reduced risk of developing chronic disease [12]. The mechanism by which carotenoids mediate this risk is not clear, nor is the contribution of individual carotenoids, but the risk reduction has typically been ascribed to anti-inflammatory and antioxidant effects [13]. The richest dietary sources of lutein are green vegetables, including spinach, kale, zucchini, broccoli, green beans and asparagus, as well as corn, pumpkin, squash, egg yolks, and fruits including kiwi and grapes [14]. Western diets where fruit and vegetable intakes are decreasing [15], or not meeting recommended intakes [16,17], are therefore typically low in lutein.

A number of cross-sectional studies have sought to identify factors associated with a high lutein intake and/or high circulating lutein concentrations. These studies indicate that high circulating lutein concentrations are associated with high levels of PA [18,19], and low serum lutein concentrations are associated with high levels of sedentary behavior [20]. However, a causal link between lutein and PA had not been proposed. Lutein is able to cross the blood-brain barrier [21] and influence cognitive processes [22] and mood [23], but effects on behavior have not previously been evaluated.

A small double-blind, placebo-controlled, randomized controlled trial, performed by our group recently showed for the first time that lutein can positively influence PA behavior. Five weeks of lutein supplementation (21 mg/day) in sedentary middle-aged and older adults (*n* = 39) increased plasma lutein concentrations by 135%, and the magnitude of increase in plasma lutein was linearly related to increases in PA and inversely correlated with time spent sedentary [24]. In a subsequent study in rats, it was shown that when lutein was added to rat chow with milk as a lipid vehicle, serum lutein concentrations were increased and the rats increased their spontaneous wheel running activity compared with controls [25]. Given that these preliminary findings were from randomized controlled trials they suggest that increasing circulating lutein concentrations via an increased dietary intake of lutein may cause an increase in PA and reduce sedentariness. Thus, the intake of a lutein-rich diet may provide a strategy for increasing PA. The mechanism by which lutein might influence PA is unclear, but it is able to cross the blood-brain barrier and has been shown in primates to accumulate in the cerebellum, frontal cortex, occipital cortex, and pons, brain areas which are associated with a range of functions, including behavior [21].

For the present study, we used data collected from two prominent studies with similar data on diet and PA: the Maine-Syracuse Longitudinal Study (MSLS) in the United States, and the Observation of Cardiovascular Risk Factors in Luxembourg (ORISCAV-LUX). The objective of the study was to examine associations between dietary intake of lutein-rich foods and levels of PA in two study sites quite disparate in terms of dietary and PA habits. As a result of the preliminary evidence that lutein may cause an increase in PA, it was hypothesized that a higher intake of lutein-rich foods, namely vegetables, fruit and eggs, would be positively associated with PA levels in both study sites, thus providing additional evidence to support increasing dietary lutein intake as a strategy for increasing PA.

## 2. Methods

### 2.1. Subjects

The study utilized data from two cross-sectional studies, the MSLS and ORISCAV-LUX, and included 2303 individuals, 972 from MSLS and 1331 from ORISCAV-LUX. Further details related to the methods of sampling for both studies appear below and in numerous publications [26,27,28,29].

#### 2.1.1. Participants in MSLS (USA)

The MSLS is a longitudinal, community-based study of aging, cardiovascular risk factors and cognitive functioning in adults, aged 23–98 years [28,29,30,31]. The MSLS was conducted in Syracuse, New York (NY), USA and its catchment area (Central NY). At initial recruitment (1975), the sole exclusions were institutionalized people, diagnosed alcoholism, and psychiatric disorders. The data for the present cross-sectional study were taken from subjects returning for the sixth study wave (2001–2006) when dietary intake measures were first obtained. Beginning with a sample of 1049 individuals, participants were excluded from the present analysis for the following reasons: missing data on diet or PA (*n* = 35), acute stroke (*n* = 28), probable dementia (*n* = 8), undergoing hemo-dialysis (*n* = 5), inability to read English (*n* = 1), and alcohol abuse after baseline (*n* = 1), leaving 972 participants. The University of Maine Institutional Review Board approved this study and informed written consent was obtained from all participants.

#### 2.1.2. Participants in ORISCAV-LUX (Luxembourg)

ORISCAV-LUX was a nationwide, cross-sectional study on the prevalence of cardiovascular risk factors among the adult population of Luxembourg, aged 18–69 years, conducted in 2007–2009. A comprehensive description of the ORISCAV-LUX survey design, sampling methods and sample have been published elsewhere [26,27]. A representative random sample of 1432 individuals, stratified by sex, age, and district of residence completed the recruitment procedure [26,27]. After data cleaning, a sample of 1331 subjects was available for analyses from the ORISCAV-LUX study. The study was approved by the National Research Ethics Committee and the National Commission for Private Data Protection, and all participants gave informed written consent.

### 2.2. Procedure

#### 2.2.1. Dietary Assessment

In the MSLS, dietary intake was assessed using the food frequency questionnaire (FFQ) component of the Nutrition and Health Questionnaire [32,33,34]. Its acceptable validity has been demonstrated by comparison with dietary recall, protein excretion, and total energy expenditure data [32]. The dietary component questions participants about their frequency of consumption of 37 foods and beverages, from six response options: “never”, “seldom”, “once a week”, “2–4 times/week”, “5–6 times/week” and “once or more/day”. The median score within each response option was used to estimate intakes per week; for example, two to four times per week was estimated at three. Individual lutein-containing foods available for analyses were: eggs, total vegetables, fresh fruit, green vegetables, other cooked vegetables, salad/raw vegetables, peas, tomatoes, and carrots.

Portion or serving sizes were not stipulated; therefore total energy was estimated in the following manner. The mean number of times each food was consumed on a weekly and then daily basis was calculated for all foods. Individual foods were categorized into six major food groups (grains, fruits, vegetables, protein foods, dairy foods, and fats/sweets/other). Total energy was therefore estimated by summing the number of servings per day of all foods and beverages [35].

In ORISCAV-LUX, dietary intake was assessed using a validated, semi-quantified FFQ, which assessed the frequency of consumption of 134 items over the previous three months [36,37]. Participants were asked how frequently they consumed one standardized portion of each food, from six response options: “never or rarely”, “1–3 times/month”, “1–2 times/week”, “3–5 times/week”, “once/day”, and “two or more times/day”. Energy and nutrient intake data, including alcohol (g/day) and total energy intake (Kcal/day), were compiled. Individual lutein-containing foods available for analyses were: eggs, total vegetables, fresh fruit, citrus fruit, berries, kiwi fruit, pear/apple, banana, plum/grapes, green vegetables, salad/raw vegetables, other vegetables, peppers, tomatoes, and carrots [38].

#### 2.2.2. Physical Activity Data

In the MSLS, PA was measured with the Nurses’ Health Study (NHS) Activity Questionnaire [39]. This questionnaire is reported to be a valid measure of long-term PA levels [40]. Participants were asked, “during the past year, what was your average time per week spent at each of the following recreational activities?” Activities included walking or hiking outdoors, jogging (slower than 10 min/ mile), running (10 min/mile or faster), bicycling, calisthenics aerobics, exercise machine use, tennis, squash, or racquetball, lap swimming, and other aerobic recreation. The range of possible values for each activity was 0 to 11 h. A MET value for each activity was assigned using the compendium of physical activities developed by Ainsworth *et al.* [41] and values used previously by the Nurses’ Health Study investigators [39,40]. The MET values for each activity were multiplied by the number of hours spent at each activity to obtain MET-hours per week for each activity. The total MET-hours per week spent at leisure time PA was obtained by summing the MET-hours for each individual activity, and then multiplied by 60 to obtain MET-minutes per week.

In ORISCAV-LUX, PA was measured using the short format International Physical Activity Questionnaire (IPAQ) [41,42], designed and validated to measure PA in large populations. Mean weekly moderate and intense PA time (in minutes per week) was calculated by multiplying self-reported time spent engaging in each with the reported number of days per week in which these activities were undertaken. These values were summed to obtain total mean PA time per week (moderate plus intense).

Poor, intermediate, and ideal health levels for PA were calculated using the American Heart Association definitions [43], where poor equates to no PA, intermediate equates to between 1 and 149 min per week of moderate intensity activity (or 1–74 min per week of vigorous intensity activity), and ideal equates to at least 150 min per week of moderate intensity activity (or ≥75 min per week of vigorous intensity activity). In terms of MET-minutes, 150 min per week of moderate intensity activity equates to 500 MET-minutes per week [44].

#### 2.2.3. Lifestyle and Health Data

Participants in both studies underwent physical and anthropometric measurements and blood tests. Standardized protocols for data collection were used. Body weight, height, body mass index (BMI), waist circumference, and blood pressure (BP) measures were assessed as described previously for both studies [26,27,28,29,30,45]. Standard assay methods were employed [28,45] to obtain fasting plasma glucose, serum triglycerides, high-density lipoprotein (HDL)-cholesterol, low-density lipoprotein (LDL)-cholesterol, total cholesterol, and C-rective protein (CRP) (all mg/dL). All participants completed self-administered questionnaires to gain information on demographic and socioeconomic characteristics, including age, sex, education (years), and smoking (cigarettes/day).

### 2.3. Statistical Analysis

According to the type of variable (continuous or categorical), general linear modelling or Chi-square tests were used to compare demographic variables, dietary intakes, and other health-related variables in the two samples (*n* = 972 for MSLS, *n* = 1331 for ORISCAV-LUX), according to PA level (poor, intermediate, and ideal).

The same analyses were then performed for the MSLS and ORICAV-LUX samples. Multiple linear regression analyses were used to evaluate relations between fruit and vegetable consumption and PA in each study. Covariates used in the regression models were selected based on the following criteria: they had to correlate significantly with both PA and fruit/vegetable intake in both samples, and had to be theoretically meaningful. The following two multivariable regression models were used:

Covariate set 1: adjusted for age, sex, education
Covariate set 2: covariate set 1 variables, plus smoking, C-reactive protein, BMI, glucose, HDL-cholesterol, and total energy intake.

Binary logistic regression analyses were performed to calculate the odds associated with less than ideal PA levels (less than 150 min per week of moderate intensity activity), according to fruit and vegetable intake. Analyses were performed for total vegetables, total fruit, as well as for individual foods common to both studies: green leafy vegetables, salad, tomatoes, carrots, other vegetables, and eggs. The same two multivariate models were employed as above. In both models, the highest intake of fruit or vegetable intake was considered as the reference group. Results are expressed as odds ratios and 95% confidence intervals (ORs, 95% CIs).

All statistical analyses were performed with PASW for Windows^®^ version 21.0 software (formerly SPSS Statistics Inc. Chicago, IL, USA); *p* < 0.05 was considered statistically significant.

## 3. Results

### 3.1. Participant Characteristics

Table 1 shows the demographic variables, dietary intakes, PA levels, and other health-related variables for MSLS and ORISCAV-LUX participants. A similar proportion of subjects from each study had poor PA levels (10%–11%), but a greater propotion in ORISCAV-LUX met ideal levels (67%) than in the MSLS (56%). Overall, weekly PA time was over two times higher in the Luxembourg sample than in the Central NY sample. In both study samples, those who had ideal PA levels also had significantly lower blood pressure, triglyceride and CRP levels, lower BMI, and higher HDL-cholesterol, compared to those who had poor PA levels. From a dietary perspective, participants with ideal PA levels also consumed significantly more fruit and vegetables than those with both poor or intermediate PA levels.

**Table 1:** Study sample characteristics according to physical activity levels in MSLS and ORISCAV-LUX studies.

Characteristic	MSLS, *n* = 972			ORISCAV-LUX, *n* = 1331		
	Physical Activity Level ^a^			Physical Activity Level ^a^		
	Poor	Intermediate	Ideal	Poor	Intermediate	Ideal
*n* (%)	102 (10.5)	326 (33.5)	544 (56.0)	150 (11.1)	281 (21.1)	900 (66.6)
Age (years)	64.2 ± 13.7	63.9 ± 13.2	61.1 ± 12.8 ^1,2^	47.2 ± 12.9	42.7 ± 13.7 ^1^	44.4 ± 12.8 ^1^
Sex (% male)	28.4	34.7	47.2	66.7	48.0	45.7
Education (% tertiary)	20.6	37.7	48.5	24.7	35.2	24.1
Physical activity (mins/week)	0	88 ± 74 ^1^	462 ± 342 ^1,2^	0	71 ± 41 ^1^	1103 ± 963 ^1,2^
Smoking (cigarettes/day)	2.5 ± 7.3	1.6 ± 5.6	1.0 ± 4.6 ^1^	4.4 ± 9.3	2.4 ± 6.6 ^1^	2.7 ± 6.8 ^1^
Systolic blood pressure (mmHg)	139 ± 24	133 ± 21 ^1^	128 ± 21 ^1,2^	134 ± 19	129 ± 18 ^1^	129 ± 18 ^1^
Diastolic blood pressure (mmHg)	73 ± 10	71 ± 10	70 ± 10 ^1^	86 ± 11	82 ± 11 ^1^	82 ± 11 ^1^
Waist circumference (cm)	101 ± 18	97 ± 16	93 ± 14	96 ± 16	89 ± 14 ^1^	89 ± 13 ^1^
Total cholesterol (mg/dL)	196 ± 43	200 ± 40	203 ± 40	209 ± 44	200 ± 41 ^1^	201 ± 40 ^1^
HDL cholesterol (mg/dL)	50 ± 15	53 ± 15	55 ± 15 ^1^	57 ± 17	62 ± 18 ^1^	62 ± 16 ^1^
LDL cholesterol (mg/dL)	114 ± 33	119 ± 34	123 ± 32 ^1^	132 ± 39	122 ± 36 ^1^	124 ± 34 ^1^
Fasting plasma glucose (mg/dL)	107 ± 2.8	101 ± 1.5	96 ± 1.2	99 ± 23	93 ± 18 ^1^	92 ± 17 ^1^
Triglycerides (mg/dL)	177 ± 162	151 ± 108 ^1^	132 ± 101 ^1,2^	134 ± 87	124 ± 130	110 ± 82 ^1,2^
Body mass index (kg/m^2^)	31 ± 7.1	30 ± 6.6 ^1^	28 ± 5.0 ^1,2^	28 ± 6.1	26 ± 5.1 ^1^	26 ± 4.7 ^1^
C-reactive protein (mg/L)	0.6 ± 0.7	0.5 ± 0.5 ^1^	0.4 ± 0.4 ^1,2^	0.4 ± 0.6	0.2 ± 0.4 ^1^	0.2 ± 0.5 ^1^
Dietary variables						
Total energy intake ^b^	13.9 ± 4.2	14.6 ± 4.2	15.1 ± 4.8 ^1^	2366 ± 973	2366 ± 879	2447 ± 943
Vegetables (servings/day)	2.3 ± 1.2	2.6 ± 1.0 ^1,3^	2.9 ± 1.2 ^1,2^	3.6 ± 2.6	3.6 ± 2.5	4.0 ± 2.8 ^2^
Fruit (servings/day)	1.3 ± 1.1	1.6 ± 0.9	1.7 ± 1.0 ^1,2^	1.5 ± 1.5	1.6 ± 1.8	1.9 ± 2.1 ^1,2^
Grains (servings/day)	3.7 ± 1.8	3.8 ± 1.8	3.7 ± 2.2	2.6 ± 1.1	2.8 ± 1.2	2.8 ± 1.2
Meat (servings/day)	1.8 ± 0.8	2.1 ± 0.9 ^1^	2.0 ± 0.9 ^1^	1.2 ± 0.7	1.2 ± 0.7	1.2 ± 0.7
Dairy foods (servings/day)	1.8 ± 1.1	1.9 ± 1.1	2.1 ± 1.1 ^1,2^	2.0 ± 1.5	2.3 ± 1.7	2.4 ± 1.6 ^1^
Alcohol (standard drinks/day)	0.3 ± 0.7	0.4 ± 0.7	0.6 ± 1.0 ^1,2^	0.9 ± 0.8	0.7 ± 0.7	0.8 ± 0.8

Values are mean ± SD unless otherwise indicated. HDL = high density lipoprotein, LDL = low density lipoprotein, MSLS = Maine-Syracuse Longitudinal Study, ORISCAV-LUX = Observation of Cardiovascular Risk Factors in Luxembourg. ^a^ Poor: No physical activity; Intermediate: 1–149 min/week moderate intensity activity (or 1–74 min/week vigorous intensity activity); Ideal: ≥150 min/week moderate intensity activity (or ≥75 min/week vigorous intensity activity); ^b^ Total energy intake: in Kcal/day (ORISCAV-LUX) and total servings/day all food groups (MSLS); ^1^ significantly different from poor group; ^2^ significantly different from intermediate group; ^3^ significantly different from ideal group (all *p* < 0.05).

### 3.2. Fruit and Vegetable Consumption and Physical Activity in MSLS

As may be seen in Table 2, in the MSLS, total vegetable and total fruit consumption were positively associated with total PA time (fully extended model, both *p* < 0.001). Green leafy vegetables, salad, tomatoes, carrots, and “other vegetables” were all significantly and positively related to PA (fully extended model, all *p* < 0.05). Consuming less than five servings per day of vegetables was associated with significantly higher odds of achieving less than ideal PA levels (OR: 2.6, 95% CI: 1.1–6.4), compared to consuming at least five servings per day (data not shown). Participants who did not consume at least one serving of salad per week also had significantly higher odds of achieving less than ideal PA levels (OR: 2.7, 95% CI: 1.6–4.8), compared to those who consumed at least one serving per week (data not shown).

Intakes of meat, dairy foods, eggs, and alcohol were unrelated to PA, while total grain intake was inversely associated with PA (data not shown).

**Table 2:** Raw (unstandardized) regression coefficients (b) and standard error (SE) summarizing associations between dietary intakes (serves/week) and physical activity (min/week) in MSLS and ORISCAV-LUX studies.

Predictor	MSLS, *n* = 972			ORISCAV-LUX, *n* = 1331		
	b	SE	*p*	b	SE	*p*
Total vegetables	6.5	1.5	<0.001	3.8	1.4	0.007
Green leafy	11.5	5.6	0.04	40.4	15.8	0.011
Salad	23.0	5.2	<0.001	31.6	10.5	0.003
Tomatoes	19.0	5.7	0.001	6.9	12.0	0.6
Carrots	20.4	6.6	0.002	17.1	16.5	0.3
Other vegetables	16.7	5.5	0.002	15.7	10.6	0.1
Total Fruit ^a^	13.4	3.0	<0.001	1.3	2.0	0.5
Plums/grapes	NA			25.3	12.2	0.04
Eggs	−3.3	3.9	0.4	−6.3	2.2	0.8

^a^ Excludes fruit juice. MSLS = Maine-Syracuse Longitudinal Study, NA = not available, ORISCAV-LUX = Observation of Cardiovascular Risk Factors in Luxembourg. Presented data are for the extended model; regression coefficients were adjusted for age, education, gender, body mass index, HDL-cholesterol, C-reactive protein, fasting plasma glucose, smoking (cigarettes/day), total energy intake. Note the same pattern of significant results with similar regression coefficients were obtined for the basic model, and thus are not shown.

### 3.3. Fruit and Vegetable Consumption and Physical Activity in ORISCAV-LUX

In ORISCAL-LUX, total vegetable consumption was positively associated with total PA time (fully extended model, *p* < 0.01). Green leafy vegetables and salad were positively associated with PA (fully extended model, both *p* < 0.05). Participants who did not consume at least one serving of salad per week also had significantly higher odds of less than ideal PA levels (OR: 1.7, 95% CI: 1.2–2.4), compared to those who consumed more than this (data not shown). Total fruit intake was not related to PA, but plum/grape consumption was positively associated with PA (fully extended model, *p* < 0.05). Total daily intake of grains, meat, dairy foods, eggs, and alcohol were unrelated to PA.

## 4. Discussion

We have demonstrated significant associations between a higher intake of lutein-rich foods, namely vegetables, and in particular green leafy vegetables, and a higher level of PA, in two different study sites. It might be argued that this association exists because people who consume more vegetables are “health conscious” and therefore also choose to engage in PA rather than being due to any effect of an increased dietary intake of lutein. However, recent data from two randomized controlled trials that supplemented the diets of rats and humans with lutein demonstrated that lutein increased PA [25] and that increases in PA were linearly related to increases in plasma lutein concentrations [24], thus suggesting a causative relationship between the consumption of lutein-rich foods and increases in PA.

The present study is also consistent with other observational research in the US [18,19]. Using data from the Third National Health and Nutrition Examination Survey [18], higher serum lutein + zeaxanthin was significantly associated with being more physically active (and with having higher dietary lutein + zeaxanthin), consistent with another US study showing a significant correlation between PA and dietary lutein + zeaxanthin intakes [19]. Correlations have also been noted between lower levels of dietary lutein + zeaxanthin and other lifestyle factors including smoking and alcohol consumption [18,19]. Several studies have also reported associations between higher levels of inflammation and lower levels of serum lutein + zeaxanthin [18,46]. However, while food frequency questionnaires that are typically used in large dietary surveys only provide assessments of the sum of lutein and zeaxanthin intakes, the increases in PA and reductions in sedentary time that were observed in our preliminary study [24] were only correlated with changes in plasma lutein and not changes in plasma zeaxanthin, suggesting that effects on PA behavior are only related to changes in lutein status.

Mean PA time was considerably higher in the Luxembourg site than Central NY, equating to a difference of approximately 6.5 h per week. This is consistent with data showing that Europeans walk and cycle, over two and four times respectively, the number of kilometres per person per year than people in North America [47]. Higher rates of walking and cycling as a means of transport have also been associated with a higher percentage of adults meeting the recommended levels of PA, as well as lower rates of diabetes [48].

We have previously shown that overall cardiovascular health, generated from seven health metrics, including diet and PA, was higher at the Luxembourg site than at the Central NY site [49]. Our findings are therefore robust, given that similar relations between lutein-rich foods and PA have been observed in two study sites that vary in terms of diet and activity levels.

Increases in physical inactivity and obesity, and decreases in fruit and vegetable consumption have been observed in the United States between 1998 and 2010 [15]. Intakes of vegetables are lower than recommended in both the United States and Australia [16,17]. Our results suggest that increasing vegetable consumption to achieve current recommended intakes of five to six servings per day for men and women aged 19–50 years [16] (or 2.5 cups of fruit and vegetables per day [17]), and including green, leafy vegetables should continue to be strongly encouraged. Modest increases in dietary lutein intake could quite readily be achieved through such dietary manipulation, providing substantial increases in plasma lutein concentrations, and potentially impacting on chronic disease risk by increasing PA. For example, increasing egg consumption to one per day in older adults has been shown to significantly increase serum lutein concentrations without elevating serum lipids and lipoprotein cholesterol concentrations, over just a five-week period [50]. The intake of eggs was not related to PA in either study site, however egg consumption was relatively low (mean intake of 3.3 eggs/week in MSLS and 1.1 eggs/week in ORISCAV-LUX). The low intakes may explain why there was no relationship observed with PA.

It is widely recognized that lutein is able to cross the blood-brain barrier and accumulates in brain regions that are associated with behavior regulation [21], and it is possible that this might contribute to the observed relationships between higher lutein intakes/blood status and increased PA. Indeed, the diet of early man included a high intake of green vegetative matter [51] and would thus have been a high-lutein diet. If the lutein from this diet accumulated in brain regions and influenced behavior to increase PA then this should have assisted with hunting and/or gathering activities, which would in turn have provided a survival advantage. Thus, from a teleological perspective, it makes evolutionary sense that the intake of lutein might influence PA behavior. However, the precise mechanism(s) by which lutein intake may positively effect PA levels remains to be ellucidated. Nevertheless, the consistent finding of an association between a higher intake of foods rich in lutein and higher levels of PA, despite the difference in PA patterns in the two study sites evaluated in the present study, adds to the robustness of the finding. We were able to examine these relationships while controlling for a number of potential confounding variables, such as cardiovascular risk factors which were collected in both studies. Further, both FFQ’s collected information about intakes for the same specific foods that were used in the analysis, enabling comparison between sites. Each questionnaire enabled intake frequencies (of the same foods) to be calculated in terms of servings per day and week. In addition, the same individual foods from both questionnaires were used to calculate the total intakes of each of the main food groups (compared in Table 1). Thus, the analysis of the dietary data was comparable across sites and, despite the differences in PA patterns across sites, in both cases higher PA levels were associated with a greater intake of foods rich in lutein.

A number of limitations should be acknowledged. We are unable to generalize beyond the two geographic study sites in Central NY, USA and Luxembourg to draw conclusions regarding the United States and Luxembourg. Our focus in this study is on the agreement in findings for two different sites in two geographically and culturally disparate environments. Diet and PA data were based on participant self-reporting and the same instruments were not used in both studies. The blood analyses were not performed in the same laboratory, albeit standardized procedures were used in both. Also, the absorption of lutein is variable and dependent to a large extent on the food matrix within which it is consumed. Thus, it would have been beneficial to be able to evaluate effects of differences in blood lutein status on PA, but unfortunately these data were not available. An association between the consumption of lutein-rich foods and PA cannot be interpreted as causative. However, the fact that two recent studies have shown that dietary lutein supplementation that results in increased circulating lutein levels, result in increased PA [25] with the magnitude of increase in PA being linearly related to the magnitude of increase in plasma lutein concentration [24], does provide some support for the proposition that an increased dietary intake of lutein might cause an increase in PA.

## 5. Conclusions

There is an urgent need to develop innovative strategies to increase PA amongst people of all ages. Altering the diet to increase lutein-rich foods may present as a novel approach for inducing PA. Randomized controlled trials are required to determine whether increases in plasma lutein concentrations as a result of an increased dietary intake of lutein can increase PA. This may help inform as to whether increased dietary intake of lutein and associated heightened levels of PA mediate improvements in risk factors for major chronic diseases, including cardiometabolic disease, cancer, dementia, and depression.
